# Growth characteristics of human parechovirus 1 to 6 on different cell lines and cross- neutralization of human parechovirus antibodies: a comparison of the cytopathic effect and real time PCR

**DOI:** 10.1186/1743-422X-10-146

**Published:** 2013-05-13

**Authors:** Brenda M Westerhuis, Sara CM Jonker, Sandhia Mattao, Kimberley SM Benschop, Katja C Wolthers

**Affiliations:** 1 Department of Medical Microbiology, Laboratory of Clinical Virology, Academic Medical Center, University of Amsterdam, Meibergdreef 15, 1105 AZ, Amsterdam, Amsterdam, the Netherlands

**Keywords:** Parechovirus, Replication, Cross- neutralization, Real time PCR, CPE

## Abstract

**Background:**

Human parechoviruses (HPeVs) are among the most frequently detected picornaviruses in humans. HPeVs are usually associated with mild gastrointestinal and respiratory symptoms with the exception of HPeV3 which causes neonatal sepsis and CNS infection. Previous studies showed various results in culturing different HPeV genotypes, inducing only a low cytopathic effect (CPE).

**Methods:**

*In vitro* growth characteristics of the different HPeV genotypes in a range of 10 different cell lines are scored with CPE and measured in the supernatant by real time PCR. In the optimal cell line for each genotype a standard neutralization assay with the available HPeV antibodies (Abs) was performed and scored by CPE and measured by real time PCR.

**Results:**

All six HPeV types were able to replicate on the RD99, A549, and Vero cell lines. HPeV1 was the only genotype able to replicate on all cell lines. Most efficient growth of HPeV1, 2, 4, 5, and 6 was shown on the HT29 cell line, while HPeV3 was unable to replicate on HT29. In all cases viral replication could be measured by real time PCR before CPE appeared. The polyclonal Abs available against HPeV1, 2, 4 and 5 all showed neutralization of their respective genotype after 7 days with inhibition of >60% in real time PCR and full inhibition of CPE, although cross-neutralization is shown. Replication of HPeV3 could only be inhibited by 12% by the anti-HPeV3 (aHPeV3) Ab and no inhibition of CPE was shown after 7 days.

**Conclusion:**

When replication is monitored by PCR, growth of HPeV genotypes 1 to 6 is supported by most of the cell lines tested, where viral replication is measured before appearance of CPE. A combination of HT29 and Vero cells would therefore support replication of all culturable HPeV types, so viral replication could be detected by PCR within 3 days for all genotypes.

In addition, we showed efficient neutralization for HPeV1, 2, 4, 5, while cross- neutralization was shown between these types, indicating possible common neutralizing epitopes. For HPeV3 no efficient (cross-) neutralization was shown, indicating different neutralizing epitopes for HPeV3 compared to the other HPeV genotypes.

## Introduction

Human Parechoviruses are single stranded RNA viruses belonging to the *Picornaviridae* family. HPeVs are associated with a wide range of clinical manifestations ranging from gastrointestinal and respiratory symptoms to more severe symptoms like central nervous system (CNS) infections and neonatal sepsis [1-4]. Nowadays, 16 genotypes are known [5-10], HPeV1 and HPeV2 were first isolated in the 1950’s, whereas the third type was only described in 2004. This late discovery of HPeV could be due to difficulties of HPeVs detection in cell culture, because of only a low induction of CPE, most pronounced for HPeV3 and HPeV6. HPeV7 to 16 have not been cultured in standard cell lines used in diagnostics. Previous studies showed various results in culturing different HPeV genotypes. HPeV1 can be cultured on various cell lines such as BSC-1, Caco2, RD-18S, Vero, LLCMK2, RD99, HT29, tMK, and A549, while other newer HPeVs induce only a low cytopathic effect (CPE) on a limited number of cell lines [7,11,12]. For isolation of HPeV1, HPeV3 and HPeV6 from clinical samples, Watanabe et al. used 8 different cell lines [7]. For HPeV3 it was shown that initial culturing of 3 clinical specimen showed induction of CPE on the LLCMK2 cell line after 14–18 days, albeit after passasing the virus to Vero cells, CPE appeared after 4–5 days [1]. Benschop et al. showed that the HT29 cell line is an efficient cell line to propagate most HPeVs from clinical samples except for HPeV3, which could only be isolated on A549 and Vero cells [12]. None of the HPeVs could be detected by growth on the HEL cell line, a cell line that is regularly used to culture enteroviruses (EVs). In addition it was shown that only 42% of clinical samples positive for HPeV by PCR could be cultured, using 6 different cell lines [12]. With difficulties in culturing HPeVs, there is also a limitation in isolation of all the different strains for usage in HPeV serotyping assays*.* Because HPeV detection with cell culture is laborious and limited to HPeV1-6, PCRs targeting the 5’UTR are commonly used to diagnose HPeV infection [12-15]. Since the 5’UTR is highly conserved all HPeV types can be detected, and this method is highly sensitive. However, cell culture is still used as a diagnostic method in laboratories worldwide. In addition, cell culture is imperative to obtain virus isolates for further studies.

Although cell culture is a commonly used method in diagnostics and research, HPeVs are difficult to culture for many laboratories (personal communication). HPeVs culture characteristics have never been completely elucidated in cell culture experiments. In this study we investigated *in vitro* growth characteristics of different HPeV genotypes by measuring viral RNA in the supernatant of a range of cell lines by real time PCR. In addition, neutralization capacities of available type-specific antibodies is tested in *in vitro* cell culture.

## Results

### Replication of HPeV1 to 6 on different cell lines

Virus growth characteristics of HPeV1 to 6 were determined on HT29, Caco-2, A549, RD99, Hep-2, Vero, BGM, LLCMK2, SK-N-SH, and SH-SY-5Y by infection with a fixed MOI (0.001). Replication was monitored in the culture supernatant at day 0, 1, 3, 7 and 10 by quantitative RT-PCR, and CPE was scored at the same time points.

**Figure 1:**
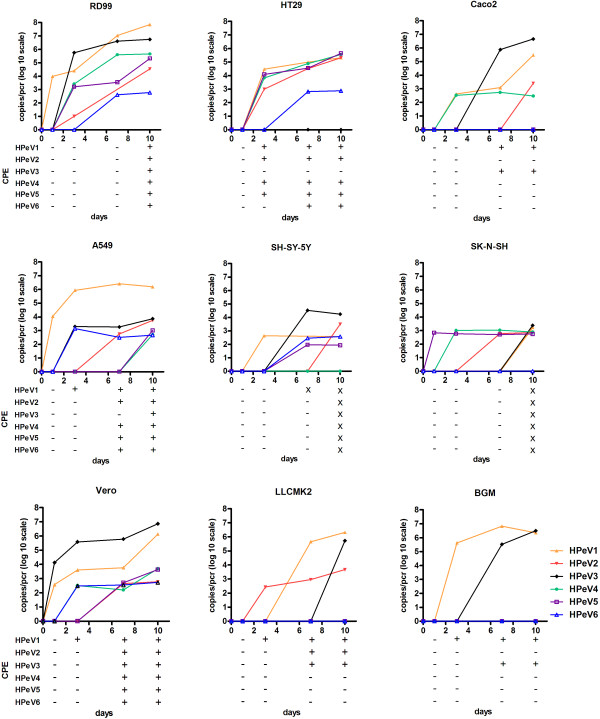
**Replication kinetics of HPeV1 to 6.** Growth kinetics of laboratory adapted HPeV1 to 6 on different cell lines including appearance of CPE at days 0, 1, 3, 7 and 10. Cells were infected with HPeVs at a MOI 0.001 and viral RNA was detected in the supernatant with RT-PCR at days 0, 1, 3, 7 and 10. The 10log virus copies were calculated with a standard curve, and the input virus copies per PCR at day 0 was subtracted. HPeV1: orange line, HPeV2: red line, HPeV3: black line, HPeV4: green line, HPeV5: yellow line, HPeV6: blue line. CPE was scored as positive (+), negative (−) and dead cells (X).

All six HPeV types were able to replicate on the RD99, A549, and Vero cell lines (Figure 1, left panel). HPeV1 was able to replicate on all cell lines. Rapid replication kinetics reaching high virus titers were found on A549, RD99 and BGM cells. Rapid replication kinetics with lower titers were found on HT29, Caco-2 and LLCMK2, while replication kinetics were slow on SH-SY-5Y and SK-N-SH cells. HPeV2 showed good replication on the HT29 cells, no replication on the BGM cell line and only slow replication kinetics on the other cell lines reaching low titers. HPeV3 reached high titers on Vero, RD99, and Caco-2 cells but could also replicate on SH-SY-5Y, SK-N-SH, LLCMK2, A549, and BGM. HPeV3 was unable to replicate on HT29, while this cell line supported the growth of all other HPeV types. HPeV1 and 3 were the only two strains able to replicate on BGM cells. Replication kinetics of types HPeV4 to 6 on the cell lines supporting their growth were mostly slower and/or reached lower virus titers than for HPeV1 or 3. HPeV4 and 5 showed good replication on the HT29 and RD99 cells, and only low replication on the A549, Caco2 (HPeV4), SH-SY-5Y (HPeV5), SK-N-SH and the Vero cells. HPeV6 showed inefficient replication: only slow replication was found in A549, RD99, Vero, HT29 and SH-SY-5Y. None of the HPeV types were able to replicate on Hep-2 cells (data not shown). On the Caco-2 cells CPE induction was limited to HPeV1 and 3, appearing in 7 to 10 days, while viral replication of HPeV2 and 4 could also been shown within 6 days by real time PCR . For HPeV3 CPE appears after 7 days only on the Vero cell line, while viral replication could be measured within one day with PCR.

Overall, replication of HPeV1 to 6 was observed in most cell lines including neural cell lines, although with different kinetics. With respect to replication on the human-derived cell lines, differences were most pronounced in the A549 with highest growth kinetics of the HPeV1, and in the HT29 where all genotypes could replicate except HPeV3. There was no cell line exclusively supporting replication of a specific HPeV genotype.

### Neutralization capacity

Polyclonal Abs available against HPeV1 to 5 were tested for neutralization capacity against HPeV1 to 6 by inhibition of CPE in cell culture (Table 1) and the inhibition was measured by real-time PCR (Table 2) at day 3 (Additional file 1: Table S1,S2) and day 7 (Tables 1,2). Only the aHPeV2 Ab fully neutralized its respective genotype without any cross-neutralization. The aHPeV1 Ab showed substantial cross-neutralization, with the HPeV4 and HPeV5 prototypes showing 60% inhibition of replication and reduced CPEs from 4+ to 2+. The aHPeV1 Ab also inhibited replication of HPeV6 by 27%, showing a reduction in CPE from 4+ to 3+. The aHPeV4 Ab inhibited replication of both HPeV4 and HPeV5 to the same extent (56-60%) with full inhibition of CPE formation, while the minor replication inhibition up to 16% for the other HPeV types was not visible by reduction of CPE. The aHPeV5 Ab showed high cross-neutralization with HPeV1 and lower with HPeV4 and HPeV6.

**Table 1:** Neutralization of HPeV1 to 6 by polyclonal Abs, read out of CPE at day 7 post infection

	**CPE score day 7**					
	**aHPeV1-Ab**^**2**^	**aHPeV2-Ab**^**2**^	**aHPeV3 Ab**^**2**^	**aHPeV4 Ab**^**2**^	**aHPeV5 Ab**^**2**^	**No Ab**
**HPeV1- Harris**^**1**^	- *	4+	4+	4+	-	4+
**HPeV2- 751312**^**1**^	4+	-	4+	4+	4+	4+
**HPeV3- 150237**^**1**^	4+	4+	4+	4+	4+	4+
**HPeV4- 251176**^**1**^	2+	4+	4+	-	1+	4+
**HPeV5- 552322**^**1**^	2+	4+	4+	-	-	4+
**HPeV6- 550389**^**1**^	3+	4+	4+	4+	2+	4+

^1^ 100TCID50 infection on HT29 (HPeV1, 2, 4, 5) and Vero (HPeV3) cells.

^2^ Ab dilution 1:100.

* - no CPE.

1+ 0- 25% CPE.

2+ 25-50% CPE.

3+ 50-75% CPE.

4+ 75-100% CPE.

**Table 2:** Neutralization of HPeV1 to 6 by polyclonal Abs, percentage of inhibition measured by real time PCR at day 7 post infection

	**Percentage inhibition in real-time PCR day7**				
	**aHPeV1-Ab**^**2**^	**aHPeV2-Ab**^**2**^	**aHPeV3 Ab**^**2**^	**aHPeV4 Ab**^**2**^	**aHPeV5 Ab**^**2**^
**HPeV1- Harris**^**1**^	85	6	7	6	84
**HPeV2- 751312**^**1**^	8	80	1	5	2
**HPeV3- 150237**^**1**^	2	0	12	5	5
**HPeV4- 251176**^**1**^	39	0	0	64	14
**HPeV5- 552322**^**1**^	58	0	0	54	63
**HPeV6- 550389**^**1**^	28	0	1	0	30

^1^ 100TCID50 infection on HT29 (HPeV1, 2, 4, 5) and Vero (HPeV3) cells.

^2^ Ab dilution 1:100.

The aHPeV3 Ab inhibited replication of our laboratory prototype HPeV3-150237 only by 12%, while no reduction of CPE was shown after 7 days. Culturing for 3 or 7 days before measuring replication by PCR did not make a difference (Additional file 1: Table S2).

## Discussion

This study shows different replication kinetics of the culturable HPeV genotypes 1 to 6 on a set of different cell lines with real time PCR. Previously, difficulties have been encountered in culturing HPeVs, especially for HPeV3 and HPeV6 [1,7,12], which could only be propagated on a limited number of cell lines showing poor production of CPE. Replication monitored by PCR shows that growth of HPeV genotypes 1 to 6 was supported by most of the cell lines tested, and by comparing replication kinetics measured by PCR and seen by CPE, viral replication can be measured before CPE appears in the infected cell line, while sometimes CPE does not occur at all. In three of the nine cell lines (Vero, RD99 and A549) all six prototypes could be propagated. All HPeV1 to 6 genotypes show replication with high viral titers on the RD99 cell line, but CPE is hardly seen. The combination of HT29 and Vero cells is suitable to detect all culturable HPeV types by CPE, where viral replication could be detected with PCR within 3 days for all genotypes. By measuring replication by PCR we showed that HPeV6 was able to replicate on 5 of the 9 cell lines tested and HPeV3 on all cell lines except HT29. In contrast, HPeV3 was able to replicate on the Caco-2 gastrointestinal cell line. This difference can be due to the fact that Caco-2 cells are known for their ability to differentiate to a more fetal-like phenotype rather than adult ileal enterocytes, resulting in different receptor expression [16,17]. Between the different genotypes, replication kinetics were found to differ between cell lines, however there was no cell line exclusively supporting replication of a specific HPeV genotype. All cell lines used in our study are continuously growing cell lines obtained from tumours that do not necessarily represent the tissue from which they originated. To get better representation of the *in vivo* replication of the different genotypes, primary cell systems need to be set up.

In our study we showed that the available HPeV Abs neutralized their respective genotype with high percentages of inhibition after 7 days. Thereby we show that inhibition of viral replication can already be measured after 3 days with similar inhibition percentages, while CPE is only starting at that time for some genotypes. Measuring neutralization by reduction of CPE or replication inhibition by PCR is comparable: inhibition of replication > 60% showed full reduction of CPE after 7 days. Inhibition between 20-60% gives reduction of 1+ or 2+ of CPE and inhibition of <20% shows no reduction of CPE. By PCR as well as by CPE read out, we showed cross neutralization of presumably type-specific polyclonal Abs, while neutralizing Abs for picornaviruses are considered to be serotype-specific. However, cross-neutralization has been reported before as shown in two studies with CAV9 and HPeV1 [18,19]. For EVs the main immunogenic region is the capsid protein VP1. In both CAV9 and HPeV1 the C-terminus of the capsid protein VP1 contains the arginine- glycine- aspartic acid (RGD), which has been shown to be an important antigenic site. Although by peptide scanning a highly immunodominant epitope has been recognized in the N-terminal region of the VP0 capsid, of which the neutralizing Abs against VP0 showed high reactivity with HPeV1. Abs raised against these different antigenic sites possibly differ in their ability to cross- neutralize HPeV infections. Reinfections in children aged 0–3 years are reported, showing a lower incidence of a second infection and infections within the first year after the initial infections were rare [20,21]. This lower incidence of a second infection in children is possibly due to partial cross- neutralization. Thereby HPeV infections in adults are hardly found.

For HPeV, the available Abs are all polyclonal obtained from immunization of animals, hence the presence of Abs that can bind to common epitopes is expected. Generating seroprevalence data this cross- neutralization should be taken in account. As we have shown before, the available aHPeV3 Ab A308-99 was not able to neutralize our HPeV3-150237 strain despite efficient binding [22]. The lack of neutralisation of HPeV3 with the Japanese Ab could indicate that the antibody has partly lost its potency. However, previously we showed that after HPeV3 infection in two different donors the obtained sera did not neutralize HPeV3. Further research is needed to confirm whether HPeV3 is indeed difficult to neutralize, or whether these results are based on *in vitro* artefacts such as a defect antibody or the influence of the cell line used for *in vitro* neutralization assays. It could be that the HPeV3 virus structure does not permit Abs to reach the neutralizing epitope, but more research is needed to elucidate the mechanism of HPeV3 neutralization.

In summary, we showed that when replication is monitored by PCR, growth of HPeV genotypes 1 to 6 is supported by most of the cell lines tested. Viral replication could be measured before CPE appeared in the infected cell line. For HPeV1, 2, 4, 5 and 6 neutralization is shown with high inhibition of viral replication; with cross neutralization shown, in contrast the available HPeV3 Ab shows almost no (cross-) neutralization. More research needs to be done to elucidate the differences between HPeV3 and the other genotypes.

## Materials and methods

### Cell lines

For virus culture, the following cell lines were used: human colon carcinoma (HT29), human colon adenocarcinoma (Caco-2), human lung carcinoma (A549, kindly provided by the University Medical Center, Leiden), rhabdomyosarcoma (RD99), epidermoid carcinoma of the larynx (Hep-2), African green monkey kidney (Vero), buffalo green monkey kidney (BGM, kindly provided by Dr. van Kuppeveld, St. Radboud University, Nijmegen), rhesus monkey kidney (LLCMK2, kindly provided by the Municipal Health Services, Rotterdam), and human neuroblastomas (SK-N-SH, kindly provided by Dr. Scheper, department of Neurogenetics, Academic Medical Center; SH-SY-5Y, kindly provided by Dr. Tauriainen, University of Tampere, Finland). The cells were maintained in Eagle’s Minimum Essential Medium (EMEM) supplemented with L-glutamic acid (0.2X), non essential amino acid (1X), streptomycin (0.1 μg/ml) and ampicillin (0.1 μg/ml). For HT29, A549, RD99, Hep-2, Vero, LLCMK2, and BGM the medium was supplemented with 8% heat-inactivated Fetal Calf Serum (FCS) and for the Caco-2 cell line with 20% heat-inactivated FCS. The human neuroblastoma cell lines were cultured in Dulbecco’s MEM and supplemented with 10% heat-inactivated FCS, L-glutamic acid (0.2X), non essential amino acid (1X), streptomycin (0.1 μg/ml) and ampicillin (0.1 μg/ml).

### Virus strains/Virus cultivation

The following HPeV strains were used as prototypes: HPeV1A Harris, HPeV2-751312, HPeV3-150237, HPeV4-251176, HPeV5-552322 and HPeV6-550389 [12,23,24]. HPeV1-Harris and the HPeV2-751312 strains were provided by the Dutch National Institute for Public Health and the Environment (RIVM), Bilthoven, the Netherlands, and passaged to obtain a sufficient virus stock. The HPeV3 to 6 were isolated from stool passaged two to three times to obtain sufficient virus stocks. HPeV1, 2, 4, and 5 were cultured in the HT29 cell line, HPeV3 in the Vero cell line and HPeV6 in the RD99 cell line and the virus working stocks were stored in aliquots at −80°C. The virus concentration was determined by the median tissue culture infective dose (TCID50) and calculated by the Reed and Muench method [25].

### Antibodies

The anti-HPeV (aHPeV) Abs against HPeV1 (Harris) and 2 (Williamson) were obtained from a rabbit Ab pool prepared at the RIVM. The aHPeV3 (A308-99) Ab was a kind gift from Dr. Shimizu, National Institute of Infectious Diseases, Tokyo, Japan, prepared as pooled guinea pig serum [10]. The aHPeV4 (S2592) and aHPeV5 (S2663) Abs were a kind gift from Dr. Schnurr, Viral and Rickettsial Disease Laboratory, California Department of Health Services, Berkeley USA, prepared as Armenian Hamster pooled serum [26].

### Virus replication curves

Monolayers of the different cell lines were cultured in 24 wells plates (Cellstar) with 1 ml medium and incubated at 37°C, 5% CO2. At day 0 the cell lines with a confluence of ~80% were infected with HPeV at a multiplicity of infection (MOI) of 0.001 in a volume of 200 μl culture medium for two hours, after which the non absorbed virus was removed and replaced with 1 ml maintenance medium (EMEM 2%, DMEM 2%) and incubated for 10 days. The low MOI is chosen to elucidate the entire process of the infection cycle of HPeV in cell culture. Twenty μl culture supernatant was removed for RNA extraction and quantitative RT-PCR detection at days 0, 1, 3, 7 and 10. The supernatant was extracted by automatic extraction using the total nucleic acid isolation kit with the MagnaPure LC instrument® (Roche Diagnostics). The RNA was eluted in 50 μl elution buffer and reverse transcribed as described previously [12]. Five μl of cDNA was used for real-time PCR using the LC480 (Roche Diagnostics) [12]. The virus copies per PCR were calculated with a standard curve as described previously [13]. The virus replication was normalized to the number of virus copies per PCR on day 0 (input virus). At day 10, supernatants were genotyped to confirm the input virus strain by VP1 genotyping as described before [13]. All replication experiments were first optimized with different MOI and all experiments were done in two-fold.

### Neutralization assay

Abs were mixed with the different HPeV1 to 6 virus suspension containing 100 TCID50/50 μl. Ab dilutions of aHPeV1, aHPeV2, aHPeV3, aHPeV4 and 5 (1:100) were used for end-point neutralization of 100TCID50 HPeV1 to 6. Mixtures were incubated at 37°C for 1 hr, and were used to inoculate HT29 cells (HPeV1, 2, 4, 5, and 6) and Vero cells (HPeV3) on a 96-wells plate (200 μl). Virus, cell and Ab controls were included as positive and negative control. The cells were examined for the appearance of CPE every 24hrs for 3 and 7 days and amount of viral copies were defined by real time PCR.

## Competing interests

The authors declare that they have no competing interests.

## Authors’ contributions

BW, KB, KW contributed to the study design. BW, SM, SJ contributed to acquisition of data, and analysis and interpretation of the data. BW, KB, KW have been involved in preparing the manuscript. All authors read and approved the final manuscript.

## Supplementary Material

Additional file 1: Table S1 Neutralization of HPeV1 to 6 by polyclonal Abs, read out of CPE at day 3 post infection. **Table S2**. Neutralization of HPeV1 to 6 by polyclonal Abs, percentage of inhibition measured by real time PCR at day 3 post infection. Click here for file
