# Supplementary material for: Growth characteristics of human parechovirus 1 to 6 on different cell lines and cross- neutralization of human parechovirus antibodies: a comparison of the cytopathic effect and real time PCR
Source: Virol J. 2013 May 13;10:146. doi: 10.1186/1743-422X-10-146 (PMC3674907; doi:10.1186/1743-422X-10-146)
Supplement: Additional file 1: Table S1 — Neutralization of HPeV1 to 6 by polyclonal Abs, read out of CPE at day 3 post infection. Table S2. Neutralization of HPeV1 to 6 by polyclonal Abs, percentage of inhibition measured by real time PCR at day 3 post infection. [file 1743-422X-10-146-S1.doc]

**Additional file 1**

**Table S1**. Neutralization of HPeV1 to 6 by polyclonal Abs, read out of CPE at day 3 post infection.

|  | **CPE score Day 3** | | | | | |
| --- | --- | --- | --- | --- | --- | --- |
|  | **aHPeV1-Ab^2^** | **aHPeV2-Ab^2^** | **aHPeV3 Ab^2^** | **aHPeV4 Ab^2^** | **aHPeV5 Ab^2^** | **No Ab** |
| **HPeV1- Harris^1^** | - * | 2+ | 3+ | 1 | - | 2+ |
| **HPeV2- 751312^1^** | 1+ | - | 1+ | 1+ | 1+ | 1+ |
| **HPeV3- 150237^1^** | 1+ | 1+ | 1+ | 1+ | 1+ | 1+ |
| **HPeV4- 251176^1^** | - | 3+ | 3+ | - | - | 3+ |
| **HPeV5- 552322^1^** | - | 3+ | 3+ | - | - | 3+ |
| **HPeV6- 550389^1^** | - | 2+ | 2+ | 2+ | - | 2+ |

^1^ 100TCID50 infection on HT29 (HPeV1, 2, 4, 5) and Vero (HPeV3) cells

^2^ Ab dilution 1:100

* - no CPE

1+ 0- 25% CPE

2+ 25-50% CPE

3+ 50-75% CPE

4+ 75-100% CPE

|  | **Percentage inhibition in real-time PCR Day3** | | | | | |
| --- | --- | --- | --- | --- | --- | --- |
|  | **aHPeV1-Ab^2^** | **aHPeV2-Ab^2^** | **aHPeV3 Ab^2^** | **aHPeV4 Ab^2^** | **aHPeV5 Ab^2^** |  |
| **HPeV1- Harris^1^** | 100 | 2 | 0 | 11 | 100 |  |
| **HPeV2- 751312^1^** | 0 | 100 | 0 | 16 | 26 |  |
| **HPeV3- 150237^1^** | 10 | 6 | 12 | 6 | 13 |  |
| **HPeV4- 251176^1^** | 60 | 0 | 0 | 60 | 47 |  |
| **HPeV5- 552322^1^** | 60 | 0 | 0 | 56 | 43 |  |
| **HPeV6- 550389^1^** | 27 | 0 | 0 | 2 | 33 |  |

**Table S2**. Neutralization of HPeV1 to 6 by polyclonal Abs, percentage of inhibition measured by real time PCR at day 3 post infection**.**

^1^ 100TCID50 infection on HT29 (HPeV1, 2, 4, 5) and Vero (HPeV3) cells

^2^ Ab dilution 1:100
